# Language disorders or mild cognitive disorder. About a case

**DOI:** 10.1192/j.eurpsy.2022.1685

**Published:** 2022-09-01

**Authors:** M. Valverde Barea, M. Solis, E. Perdiguero Sempere, M. Ortigosa Luque, J. Santiago Paris

**Affiliations:** 1 Universitary Hospital of Jaén, Psychiatric, Jaén, Spain; 2 UHJ, Psychiatric, Granada, Spain

**Keywords:** language impairment, memory disorder, cognitive disorder, Depression

## Abstract

**Introduction:**

Patients with mild cognitive impairment may present deficits in naming, speech production, oral comprehension and written comprehension. In the differential diagnosis, cerebrovascular disease that can lead to cognitive impairment must also be differentiated from endogenous depressive disorder or language impairment.

**Objectives:**

The aim is to highlight the importance of differential diagnosis in cognitive disorders in relation to a case.

**Methods:**

A 68-year-old female patient attended a psychiatric consultation derived from neurology when presenting a language disorder. The husband who accompanies her and the patient indicate that she has problems finding words and substitutes other expressions for them or sometimes does not answer or does so with something different from the topic that is being asked. She refers that she presents repetitive language with memory problems, alteration in the evocation of memories. The patient reports mood swings and irritability and crying with a low tolerance for frustration since she cannot express herself. Cranial MRI: cortical and central involutional changes. Periventricular leukoaraiosis and ischemic gliosis-like lesions in the white matter of both hemispheres. Psychopathological exploration: Conscious, oriented. She smiles at the questions but doesn’t answer them. Repetitive language. Alteration in the articulation of language. Depressed mood reactive to current situation. Some irritability Alteration in recent memory and evocation.

**Results:**

She was diagnosed with organic mental disorder compatible with mild cognitive impairment. Treatment with rehabilitation of the language disorder of vascular etiology is established.

**Conclusions:**

Imaging and neuropsychological tests should always be performed in a patient with language, memory, and mood disorders to study its etiology.

**Disclosure:**

No significant relationships.

